# Targeting Senescent Cells as Therapy for CKD

**DOI:** 10.34067/KID.0000000000000316

**Published:** 2023-12-05

**Authors:** Katie J. Mylonas, David A. Ferenbach

**Affiliations:** Centre for Inflammation Research, Institute for Regeneration and Repair, University of Edinburgh, Edinburgh, UK

**Keywords:** cell ablation, cell survival, CKD, fibrosis, progression of renal failure

## Abstract

Senescent cells accumulate in the kidney with aging, after acute and chronic injuries, and are present in increased numbers in deteriorating kidney transplants. Senescent cells have undergone permanent cell cycle arrest and release many proinflammatory cytokines/chemokines and profibrotic factors: the senescence-associated secretory phenotype. Recent work from several groups including our own has shown that senescent cells play a causative role in progression of kidney disease. Experimental evidence also indicates that targeting senescent cells has potential to alter the renal regenerative response, reducing progressive fibrosis and improving functional recovery after injury. Research and clinical interest is focused on understanding how accumulating chronic senescent cells link acute injury to progressive fibrosis, dysfunction, and mortality in human CKD. In this review, we outline current protocols for the identification of how senescent cells are identified *in vitro* and *in vivo*. We discuss the proposed mechanisms of actions of first-generation senolytic and senomorphic agents, such as ABT-263 (navitoclax) which targets the BCL2 family of survival factors, and senomorphic agents such as metformin which targets aspects of the senescence-associated secretory phenotype. We also review that emerging technologies, such as nanocarriers, are now being developed to have safer delivery systems for senolytics, greater specificity, fewer off-target effects, and less toxicity. Other methods of senescent cell elimination being developed target various immune evasion tactics displayed by these cells. By understanding the role of senescence in kidney homeostasis and disease, developing new, targeted compounds and the tools to allow their efficacy to be charted noninvasively, it should become possible for senolytic treatments to move from the bench to bedside.

## Introduction

The US Census Bureau predicts that by 2030, one in five Americans will be 65 years or older. This aging demographic is at the elevated risk of various diseases, including AKI, CKD, and diabetes, with associated diabetic nephropathy (DN).^1^ The likelihood of having two or more significant clinical conditions (comorbidities) also increases with age, *e.g.*, kidney disease and heart disease.^2^ Therefore, this predicted increase in people older than 65 years will lead to a substantial increase in demand for health services.

## Cellular Senescence

**Figure 1:**
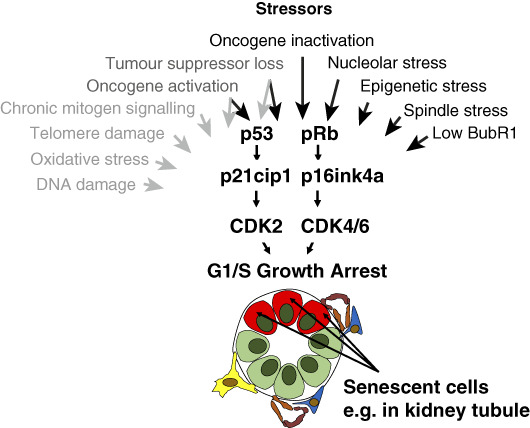
**Multiple stressors can induce cellular senescence *via* upregulated expression of the cyclin-dependent kinase inhibitors p21cip1 and p16ink4a.** These factors lead to growth arrest at the G1/S cell cycle checkpoint.

The word “senescence” comes from the Latin word for old and was originally used by biologists to describe the decline in key biologic functions over time with aging.^3^ However, from 1961, when Hayflick and Moorhead described how fibroblasts in culture had a finite capacity for replication after serial passage,^4^ cellular senescence came to describe a cellular process or cell fate, whereby cells adopted a state of irreversible cell cycle arrest but remained viable.^5^ These senescent cells also produced a secretome termed the senescence-associated secretory phenotype (SASP).^6^ It is now known that cellular senescence can be caused by a number of stressors, including DNA damage, mitochondrial dysfunction, oncogene activation, oxidative stress, epigenetic changes, mechanical stresses, factors secreted by other senescent cells, and many other mechanisms (Figure 1).^7–11^ These stressors activate DNA damage response signaling which results in the induction of the cyclin-dependent kinase inhibitor p16INK4a, and/or p21Cip1, activated by the transcription factor p53. These induce cell cycle arrest at the G1/S cell cycle checkpoint (Figure 1).^7,12–14^

Despite the accumulation of damage signals that can lead to apoptosis (*e.g.*, DNA breaks, upregulation of the DNA damage response due to reactive oxygen species production), senescence is associated with the upregulation of prosurvival and antiapoptotic pathways, *e.g.*, SRC proto-oncogene, non-receptor tyrosine kinase (SRC) kinases, the phosphoinositide-3-kinase–Protein kinase B signaling pathway, or antiapoptotic BCL-2–related proteins, all of which promote cell survival.^15–17^ Senescence induces major metabolic changes, with higher oxygen consumption and extracellular acidification rates^18^ but reduced fatty acid oxidation capacity reported in senescent cell cultures *in vitro.*^19^

## Senescence in Kidney Disease

CKD affects around 700 million people worldwide.^20^ CKD is incurable, with progressive renal fibrosis, a feature common to multiple different kidney diseases. Survivors of AKI exhibit a markedly increased risk of developing progressive fibrotic CKD after resolution of acute, self-limiting renal insults,^21^ with older patients at particular risk.^22^ It is now recognized that long-lived senescent epithelial cells accumulate in the kidney with aging, after acute and chronic injuries,^23^ and are present in increased numbers in deteriorating kidney transplants.^24^

However, levels of cellular senescence are not routinely quantified or reported on kidney biopsy analysis, and no noninvasive biomarkers, metabolic profiling,^25^ or imaging modalities can quantify the numbers of senescent cells present in the healthy, aged, or fibrotic human kidney. In the research setting, identification of altered levels of senescence markers, including p16ink4a, p21Cip1, and reduced levels of Lamin B1, can all be detected by immunofluorescence on kidney biopsies—with altered nuclear and mitochondrial morphology, blebbing, and the presence of elevated quantities of galactosidase crystals detectable on electron microscopy.

Multiple cell types in the kidney have been reported to express the markers of senescence. Previous work from the Melk laboratory^26^ showed that in aged rats, p16 staining rose in tubules, glomeruli, and interstitium, but senescence-associated beta-galactosidase (SA-*β*-gal) staining (a marker of an altered cellular phenotype) was only present in tubular epithelia consistent with these being a key site of functionally significant senescence. The same group analyzed human renal transplant biopsies, showing p16ink4a staining in the nuclei of distal tubules and collecting duct with staining also present in podocytes, parietal epithelium of glomeruli, vascular smooth muscle cells, and interstitial cells.^24^ Other work in human glomerular disease showed p16ink4a staining in a subset of glomerular, tubular, and interstitial cell nuclei; however, senescent tubular epithelial cells were an important factor differentiating diseased and control kidneys present in 80% of diseased kidneys compared with 21% in normal controls.^27^ Studies have shown that senescent glomerular endothelial cells signal to and promote podocyte apoptosis via plasminogen activator inhibitor-1-mediated signaling.^28^

**Figure 2:**
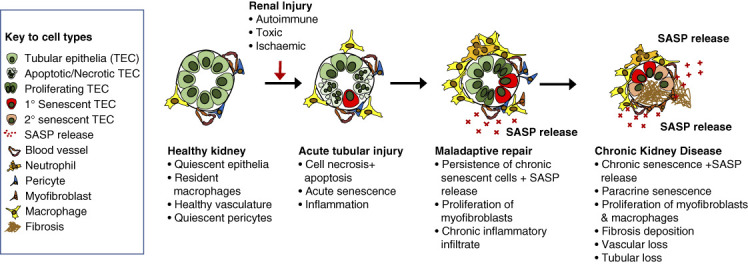
**Proposed roles for senescent cells in the evolution of maladaptive repair after acute and chronic renal injuries.** SASP, senescence-associated secretory phenotype.

Experimental work to date has focused on whether the increased numbers of senescent cells with age and disease merely represent a biomarker of aging or previous injury or whether senescent cells actively promote maladaptive repair, organ fibrosis, and dysfunction and hence represent a novel therapeutic target. Baker *et al.* demonstrated that selective senescent cell depletion *via* a senescence-activated transgene increased murine lifespan in naturally aged mice and preserved kidney health.^29^ Experimental work from our own laboratory and other groups^30–32^ has demonstrated a role for senescent epithelial cells in the evolution of kidney disease (Figure 2). This indicates that targeting senescent cells has the potential to improve the renal regenerative response, reducing progressive fibrosis and improving functional recovery after injury.

## Physiologic Functions of Senescent Cells

In embryogenesis/development, senescent cells are required for body axis patterning, with the associated SASP directing growth of structures, such as the limb bud.^33^ Senescent cells are important for postinjury repair and successful scar formation, and induction of fibroblast senescence controls fibrotic pathways required for normal healing of the skin.^34,35^ Senescence induction by oncogene activation is also recognized as an important physiologic mechanism of the body's defense against cancer.^36^

## Identification of Senescent Cells

**Figure 3:**
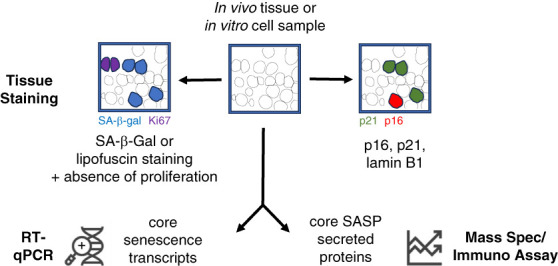
**The workflow proposed by the International Cellular Senescence Association for the identification of senescent cells from biologic samples.** SA-*β*-gal, senescence-associated beta-galactosidase. This figure was adapted from refs. 38 and 72, which should be referred to for additional protocol details.

In 2019, the International Cell Senescence Association presented a consensus statement for the identification of cellular senescence.^37^ This statement^37,38^ proposed a first staining step to identify markers differentially expressed in senescent cells—SA-*β*-gal or lipofuscin, alongside costaining for an absence of markers of proliferation. A second step was to identify an increased expression of the key cyclin-dependent kinase inhibitors p16ink4a and/or p21cip1, reduced expression of lamin B1, and alteration in the levels of core senescence transcripts. A third step included the assay of multiple core SASP-secreted proteins (Figure 3). It is important to note that no single parameter above can definitively identify a senescent cell—with macrophages expressing high levels of *β*-galactosidase in health, and multiple tissue types upregulating p16ink4a and p21cip1—particularly in the context of cellular proliferation.

Although not included in the 2019 definition, the advent of single-cell resolution RNA sequencing has advanced our ability to detect transcriptional differences in senescent cells significantly in recent years. The ability to build a “transcriptional phenotype” based on the alteration of multiple mRNA expression levels allows better analysis, clustering, and identification of healthy and senescent cells and their anatomic site of origin.^39,40^ Recently developed technologies combining single-cell resolution analysis while retaining their spatial context have the potential to advance the field further with an increasing need for computational tools to maximize the yield, interpretation, and robustness of data from each study performed.^41,42^

## Senescence-Associated Secretory Phenotype (SASP)

The SASP includes proinflammatory cytokines, matrix metalloproteases chemokines, growth factors, microRNAs, and small-molecule metabolites (Table 1),^43^ and the expression of these is largely under the control of transcription factors, such as p53, nuclear factor kappa-light-chain-enhancer of activated B cells (NF-*κ*B), and Janus kinase–signal transducer and activator of transcription. SASP components have varied functions but are typically associated with the chronic inflammatory state of aging and disease.^6^ SASP chemokines such as chemokine (C-C motif) ligand 2 (membrane cofactor protein-1) and cytokines such as IL-1, IL-8, and TNF*α* recruit immune cells, including macrophages, neutrophils, and T cells, potentially allowing for senescent and damaged neighboring cells to be located and destroyed.^44^ However, age-associated losses in immune competency, as well as immune evasion strategies, in a chronically inflamed environment may allow senescent cells to escape immune removal and persist in the tissue. Through the release of SASP factors, senescence can modulate pathways in neighboring cells and tissues as well as at remote sites. In addition, senescent cells that are induced by different stress stimuli may manifest distinctive SASP components.

**Table 1:** Reported components of the senescence-associated secretory phenotype and their links to p53 induction and oncogenic Ras

SASP Factors^a^	Secretory Profile of Senescent Cells^b^	Changes in the SASP due to the Loss of p53 and/or Gain of Oncogenic RAS
Soluble factors		
**IL**		
IL-1a, IL-1b, IL-6, IL-7, IL-13, IL-15	↑	↑
**Chemokines (CXCL, CCL)**		
IL-8, MCP-2, MIP-1a, GRO-a, GRO-b, GRO-g, Eotaxin-3	↑	↑
Eotaxin, TECK, ENA-78, I-309	×	↑
HCC-4, MCP-4, MIP-3a	↑	×
I-TAC	×	↓
**Other inflammatory factors**		
GM-CSE	↑	↑
IFN-*γ*, BLC	×	↑
MIF	↑	↓
**Growth factors and regulators**		
Amphiregulin, epiregulin, heregulin, HGF, VEGF, angiogenin, SCF, PIGF	↑	×
IGFBP-2, IGFBP-3, IGFBP-4, IGFBP-6, IGFBP-7	↑	↑ or ×
EGF, SDF1	↑ or ×	↑
bFGF, KGF (FGF7)	↑	↑
NGF	×	↓
**Proteases and regulators**		
MMP-1, MMP-3, MMP-10, MMP-12, MMP-13, MMP-14	↑	↑ or ×
TIMP-1	↓ or ×	×
TIMP-2, PAI-1, PAI-2; tPA; uPA, Cathepsin B	↑	×
**Soluble or shed receptors or ligands**		
ICAM-1, ICAM-3, sTNFRI	↑	×
OPG, uPAR, SGP130	↑	↑
TRAIL-R3, Fas, sTNFRII, EGF-R	↑	×
**Nonprotein soluble factors**		
PGE2, nitric oxide	↑	−
Reactive oxygen species	Altered	−
**Insoluble factors (ECM)**		
Fibronectin	↑	−
Collagens, laminin	Altered	−

RAS, rat sarcoma virus; CXCL, chemokine (C-X-C motif) ligand 1; MCP, membrane cofactor protein; MIP, macrophage inflammatory protein; GRO, growth-related oncogene; TECK, thymus-expressed chemokine; ENA, C-X-C motif chemokine 5; HCC, Chemokine (C-C motif) ligand 16; I-TAC, Interferon–inducible T Cell Alpha Chemoattractant; GM-CSF, Granulocyte-macrophage colony-stimulating factor; BLC, B lymphocyte chemoattractant; MIF, Macrophage migration inhibitory factor; HGF, hepatocyte growth factor; VEGF, vascular endothelial growth factor; SCF, stem cell factor; PIGF, placental growth factor; IGFBP, insulin-like growth factor-binding protein; EGF, endothelial growth factor; SDF, stromal cell-derived factor; bFGF, basic fibroblast growth factor; KGF, Keratinocyte growth factor; FGF7, Fibroblast Growth Factor 7; NGF, nerve growth factor; MMP, matrix metalloproteinase; TIMP, tissue inhibitor of metalloproteinases; PAI, plasminogen activator inhibitor; tPA, tissue-type plasminogen activator; uPA, urokinase-type plasminogen activator; ICAM, intercellular adhesion molecule; sTNFR, soluble TNF receptor; OPG, osteoprotegerin; uPAR, uPA receptor; SGP130, Soluble gp130; TRAIL, TNF-related apoptosis-inducing ligand; PGE2, prostaglandin E2; ECM, extracellular matrix.

a Factors are arranged by family.

b The secretory changes that occur at senescence are indicated by upward arrows (increase), crosses (no change), and downward arrows (decrease). Loss of p53 or gain of oncogenic RAS increases (upward arrows) or decreases (downward arrows) the secretion of several SASP factors.

Adapted from ref. 43.

## Therapeutic Targeting of Senescent Cells in Kidney Disease

**Figure 4:**
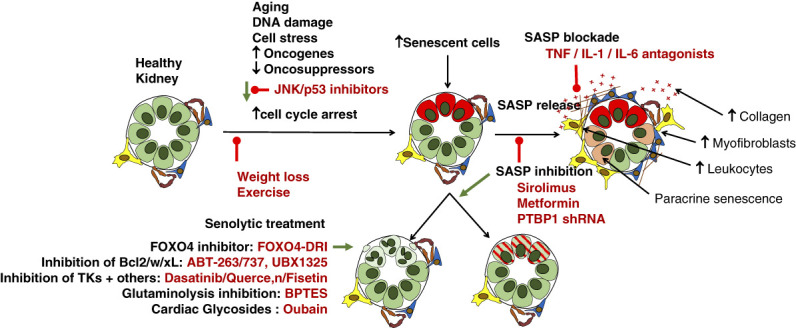
**Current and potential future invention sites to target senescent cells in the kidney *in vivo.*** B-cell lymphoma-2, BCL2; FOXO4-DRI, Forkhead box protein O4 D-Retro-Inverso. Adapted from ref. 3.

Examples of various routes proposed to tackle the pathologic effects of senescence in the kidney are presented in Figure 4. Experimental data are lacking for the impact of senescence on proteinuric kidney disease, but experimental models of CKD clearly link senescence to increasing renal fibrosis and declining eGFR over time.

## Senolytics Targeting Antiapoptosis or Prosurvival Mechanisms in Preclinical Models

Current senolytics interfere with antiapoptotic and/or prosurvival signals in senescent cells. These senolytics have been tested in several preclinical models of aging and disease, including type 2 diabetes and kidney disease.

### FOXO4-DRI

Baar *et al.* found that a molecule called FOXO4 is elevated in senescent cells and maintains their viability by repressing apoptosis through its interaction with p53.45. They designed a FOXO4 peptide (Forkhead box protein O4 D-Retro-Inverso [FOXO4-DRI]) that perturbed the FOXO4 interaction with p53, causing p53 nuclear exclusion in senescent cells. This caused selective p53 nuclear exclusion and cell intrinsic apoptosis in senescent cells. The authors found that it restored fitness and renal function in both fast aging and naturally aged mice.^45^

### Fisetin

Fisetin is a naturally occurring flavonoid with diverse mechanisms of action on multiple molecular targets and signaling pathways, including BCL-2, PI3K/AKT, p53, and NF-*κ*B. In naturally aged mice (22–24 months) and progeroid mice, fisetin treatment extended median and maximal lifespan and decreased cellular senescence in organs, including the kidney, as well as decreasing circulating SASP factors.^46^ In a model of murine lupus nephritis, fisetin treatment reduced renal senescent cell burden and fibrosis.^47^ Given its safety, fisetin is a good candidate for clinical trials in kidney disease.

### Dasatinib and Quercetin (D&Q)

Quercetin is a dietary flavonoid that is thought to selectively eliminate senescent cells by inhibiting PI3K, inducing their apoptosis.^48^ Dasatinib induces apoptosis in senescent cells by inhibiting the Src tyrosine kinase. The D+Q combination exerts broad spectrum senolytic activity through interference with several prosurvival networks, including ephrin dependence receptor signaling, phosphoinositide-3-kinase–Protein kinase B, and B-cell lymphoma-2 (BCL2) members.

Obese mice fed a high fat diet developed impaired kidney function with an upregulation of renal senescence and SASP markers. Quercetin administered for 10 weeks reduced senescence and improved function in the kidneys of these mice, as measured by decreased plasma creatinine levels.^48^ In a mouse model of ischemic nephropathy, treatment with both D&Q reduced renal senescence and damage and improved kidney function.^31^ D&Q also reduced senescence and renal fibrosis in ischemia reperfusion models of AKI and cisplatin treatment models and is now the subject of clinical trial investigation in CKD.^32^

### ABT-263

ABT-263, also known as navitoclax, inhibits BCL-2 family members upregulated in senescent cells and contributing to their apoptosis resistence.^49^ These molecules, BCL-2, BCL-XL, and BCL-W, inhibit the activation of Bax and Bak, which, in turn, mediate the permeabilization of the mitochondrial outer membrane. This pore formation allows cytochrome *c* release from the mitochondrion and downstream caspase activation which drives apoptosis. Therefore, by promoting the activation of Bax and Bak, ABT-263 promotes apoptosis of senescent cells.^50^

Depletion of senescent cells with ABT-263 reduced irradiation-induced premature hematopoietic aging and rejuvenated aged tissue stem cells.^51^ ABT-263 treatment of aged mice, and mice prematurely aged with nonlethal irradiation augmented kidney repair, limited scarring and preserved kidney tissue and function after subsequent AKI.^30^ ABT-263 treatment also reduced senescent cell load and reduced fibrosis in murine kidneys treated with cisplatin.^52^ It is important to note that the clinical use of ABT-263 was limited by thrombocytopenia.^53^ More targeted options with fewer side effects are needed to use this drug safely (see below; second-generation senolytics).

### Glutaminolysis Inhibition

Research work is ongoing to identify potential targetable pathways within senescent cells. Recent work demonstrated that lysosomal damage within many senescent cells led to the induction of kidney-type glutaminase expression to prevent intracellular acidosis.^54^ Blockade of kidney-type glutaminase function resulted in selective elimination of senescent cells and reduced markers of aging-associated tissue fibrosis and dysfunction in multiple organs, including the kidney.^54^

### Induction of Ferroptosis

Ferroptosis is an alternative, iron-catalyzed mode of cell death distinct from apoptosis. Recent work showed that senescent renal epithelial cells were sensitized to ferroptosis *via* the induction of lipooxygenase-5 and reduction in antiferroptotic glutathione peroxidase 4.^55^ The use of the ferroptosis-induced RAS-selective lethal 3 selectively eliminated senescent cells from *in vitro* cultured slices of aged kidneys and in an *in vivo* model of transplantation.

## Targeting the SASP

Senomorphics are drugs that inhibit elements of the senescent phenotype without removing the cells. Examples are discussed below.

### NF-*κ*B Inhibiting Compounds

NF-*κ*B is a transcription factor involved in inflammation, a major SASP regulator, and is the transcription factor most commonly associated with aging in a cross-species study. In a rodent model of CKD, the administration of the inhibiting compound pyrrolidine dithiocarbonate reduced renal interstitial fibrosis.^56^ The NF-*κ*B inhibitor parthenolide decreased inflammation and renal injury in cisplatin-induced renal damage models^57^ and reduced renal inflammation in unilateral ureteric obstruction models.^58^ Parthenolide demonstrated antifibrotic effects in patients with DN^59^ and glomerulosclerosis.^60^

### Metformin

Metformin is a drug commonly used to lower blood glucose levels in patients with type 2 diabetes.^61^ It also has anti-inflammatory and cardioprotective effects.^62^ Metformin affects multiple signaling pathways linked to aging, including activating adenosine monophosphate‐activated protein kinase,^63^ which inhibits inflammatory responses in diverse types of cells and tissues. Metformin inhibits the transcription factor NF-*κ*B by preventing its nuclear translocation.^64^ In a rat model of nondiabetic CKD, metformin protected kidney function, with proteomic analysis revealing reduced levels of cellular senescence.^65^ Metformin also attenuated calcineurin inhibitor-induced renal fibrosis in rats.^66^ However, metformin-associated lactic acidosis can cause metabolic acidosis in patients with eGFR <40 ml/min, and this has been shown to have a deleterious effect on renal function, limiting clinical use in patients with advanced CKD.^67^

### JAK/STAT Inhibition

The JAK/STAT pathway plays a vital role in controlling cytokine production,^68^ and as SASP molecules commonly comprise proinflammatory cytokines and chemokines, JAK/STAT inhibition is an attractive candidate for targeting with senomorphics. Aged rats have increased the levels of activated JAK1 and JAK2.^69^ Treatment of senescent cells *in vitro* with JAK inhibitors suppressed the mRNA levels of key SASP components, including IL-6, IL-8, and matrix metalloproteinase3, but did not affect the control of nonsenescent cells. Similarly, aged mice treated with a selective JAK1/2 inhibitor reduced the levels of cytokine expression compared with control animals.^69^ Janus kinase–signal transducer and activator of transcription signaling is altered in human CKD and animal models of CKD.^70^ Enhanced expression and augmented activity of JAKs and STAT3 promoted DN and their inhibition reduced disease.^71^

### PTBP1 Inhibition

An RNA-mediated interference screen revealed that targeting alternative splicing in senescent cells may be a viable approach for inhibiting the SASP. Georgilis *et al.* used a RNA-mediated interference-based approach to identify polypyrimidine tract binding protein 1 (PTBP1) as a factor mediating the inflammatory effects of the SASP. The inhibition of PTBP1 reduced the proinflammatory properties of senescent cells in the liver.^72^

### Disulfide Isomerase Family: A Member 3 (PDIA3) Inhibition

Recent work showed that PDIA3 was upregulated in senescent cells and augmented TGF-*β*–mediated fibroblast activation. The inhibition of PDIA3 *in vivo* significantly reduced kidney fibrosis during renal injury and represents a potential senomorphic therapy.^40^

## Clinical Trials of Senolytics/Senomorphics

On the basis of promising results in preclinical models, over 20 clinical trials of senolytic therapies are completed or ongoing for different diseases, some of which involve kidney disease (Table 2). Although no agents are currently licensed as senolytics or senomorphics, several classes of drugs licensed in humans have actions potentially capable of modifying SASP signaling in human CKD, with the goal of slowing or preventing eGFR loss over time (Table 3).

**Table 2:** Completed, ongoing, and planned clinical trials of senolytics in human disease

Senolytic	Trial Stage	Indication
Dasatinib+Quercetin	Phase I/II	CKD (NCT02848131), skeletal health (NCT04313634), Alzheimer disease (NCT04063124), obesity (NCT05653258)
ABT-263 (navitoclax)	Phase I/II	Cancer (NCT00445198, NCT02591059, NCT02520778, NCT02079740)
UBX0101	Phase II	Osteoarthritis of the knee (NCT04129944)—negative outcome
UBX1325	Phase II	Diabetic eye disease (NCT04857996)—positive outcome
Fisetin	Phase II	Skeletal health (NCT04313634) Sepsis (NCT05758246)

**Table 3:** Clinical agents with potential mechanisms affecting on senescent cell signaling *in vivo*

Senomorphic	Clinical Status	Indication
Metformin	Approved	Type II diabetes
Sirolimus	Approved	Immunosuppression
Ruxolitinib	Approved	Graft versus host disease
TNF blockers	Approved	Autoimmune diseases
IL-1a/b/R blockers	Approved	Autoimmune diseases
IL-6/6R blockers	Approved	Autoimmune diseases

## Second-Generation Senolytics

Senolytics are under development to have safer delivery, greater specificity, fewer off-target effects such as thrombocytopenia, and less toxicity. The examples given below involve adaptations to one first-generation senolytic, ABT-263 (navitoclax), but can also be expanded to other agents capable of inducing senescent cell death.^73^

### Nanocarriers

Nanocarriers are nanosized materials (diameter 1–100 nm) developed to carry drugs. Drugs packaged in nanocarriers comprised liposomes, micelles, or polymeric nanoparticles and offer several advantages over free drugs.^7^ They have the ability to protect the drug from premature degradation and allow more specific delivery, *e.g.*, to senescent cells. Nanocarriers conjugated with galacto-oligosaccharides have been produced for preferential delivery into SA-*β*-gal+ senescent cells. ABT-263 (navitoclax) has been encapsulated in a nanocarrier and a senolytic nanoparticle, Gal-NP(nav), generated.^74^ This was taken up by cells *via* endocytosis and endosomes and then fused with lysosomal vesicles. SA-*β*-gal enzyme activity (hydrolyzation of the *β*-glycosidic bond formed between a galactose and its organic moiety) hydrolyzed the galacto-oligosaccharide coat of the nanocarrier, releasing ABT-263 to kill senescent cells, while normal SA-*β*-gal negative healthy cells remained unaffected.^74^

### Galactose-Based Prodrugs

The enzymic activity of SA-*β*-gal has been used to produce senolytic “prodrugs.” For example, Nav-Gal, with galactose, covalently attached to ABT-263.^75^ This is preferentially taken up by senescent cells, resulting in the release of active ABT-263 and selective destruction. Nav-Gal has been found to have less thrombocytopenia than its parent drug ABT-263.^75^

### Proteolysis-Targeting Chimeras (PROTACs)

These are highly specific drugs that degrade particular proteins of interest in cells, *e.g.*, BCL2 family members. They have two parts connected by a linker. On one side is a molecule that binds a specific target protein and the other part recruits E3 ubiquitin ligase. Different cells may have different forms of E3 ubiquitin ligase. The PROTAC brings the target protein into close contact with this enzyme, which labels it with an ubiquitin tag, making it a target for degradation by the ubiquitin–proteasome system in the cell.^76^ The protein of interest is then eliminated from the cell. A PROTAC called PZ15227 has been generated by tethering ABT-263 to a cereblon E3 ligand that is expressed minimally in normal platelets. PZ15227 showed an increased ability to clear senescent cells compared with its parent drug ABT-263 with less toxicity to platelets.^77^

## Other Potential Therapies for Kidney Disease: Immune-Based Clearance

Other strategies for decreasing age-related senescent cell burden and associated pathologic conditions involve modulating immune clearance of senescent cells. These immunotherapies exploit cell surface proteins expressed at high levels on senescent cells compared with healthy cells, recently described as “the senescent surfaceome.”

### Chimeric Antigen Receptor (CAR) T Cells

A senescent surfaceome protein highly expressed on senescent cells is urokinase-type plasminogen activator receptor, associated with extracellular matrix remodeling. Amor *et al.* were able to selectively target urokinase-type plasminogen activator receptor–expressing senescent cells *in vitro* and *in vivo* using cytotoxic CAR T cells.^78^ CAR T-cell–mediated clearance of senescent cells led to better outcomes in two murine models of liver fibrosis, suggesting the feasibility and potential of this clearance strategy.^78^ However, supratherapeutic CAR T-cell dosing was associated with side effects, including hypothermia and weight loss.^79^

## Antibody Based

### Anti-CD47 Antibodies

CD47 is upregulated on senescent cells and has been hypothesized to function as a signal preventing phagocytosis by monocytes/macrophages, by binding to SIRP*α*. Therefore, CD47 represents a potential target for senolytic therapy, as blocking the interaction between CD47 and SIRP*α* should enhance macrophage clearance of senescent cells. Magrolimab, an anti-CD47 antibody, has shown favorable results in clinical research on cancer immunotherapy, inducing tumor phagocytosis and eliminating leukemia stem cells.^80^ Such therapies could be repurposed to target senescence in the kidney.

### Antiprogrammed Cell Death Protein 1 (PD-1) Antibodies

Recently, Wang *et al.* found that senescent cells have increased expression of programmed death-ligand 1 (PD-L1), in both humans and mice. PD-L1 expression correlated with higher SASP levels^81^ and prevented cell destruction by PD-1 expressing T cells. An anti-PD-1 antibody reduced the numbers of senescent cells and improved several age-related phenotypes, including signs of frailty and decreased liver function.^81^ Therefore, this strategy of enhancing immune clearance of senescence cells is another promising method to target renal senescent cells.

### Antibody–Drug/Cell Conjugates

Antibody–drug conjugates are monoclonal antibodies attached to cytotoxic drugs. Β2–acroglobulin is a recently identified membrane marker of senescence^82^ involved in the presentation of peptide antigens to the immune system. Poblocka *et al.* created an antibody–drug conjugate by conjugating a Β2–acroglobulin IgG1 monoclonal antibody with duocarmycin, an irreversible DNA alkylating agent. This drug eliminated senescent cells by releasing duocarmycin into them, whereas nonsenescent cells were not affected.^83^ Another group delivered Kidney Injury Molecule-1 conjugated mesenchymal stromal cells to kidneys in the aftermath of experimental murine renal artery stenosis induction, demonstrating reductions in longer-term senescence burden and improved GFR.^84^

## Future Challenges and Perspectives

As summarized above, major efforts are currently focused on the discovery and clinical translation of senescent cell targeting therapies. Several key questions remain unanswered, and challenges remain before senolytic or senomorphic agents reach routine clinical use in CKD.

### Which Compound Has the Best Combination of Efficacy and Safety for Any Given Disease?

- Development of new compounds with less deleterious side effects than current first-generation senolytic agents.

### Which Diseases and Patients Are Likely to Benefit from Senescence Targeting Therapies?

- Analysis is needed of large, human datasets to link senescent cell burden to clinical outcomes beyond well-established risk factors, such as eGFR, age, BP, and proteinuria.
- Development and validation of methods to identify patients with high senescent cell load (*e.g.*, renal biopsy staining).

### When Is the Optimal Time to Deliver Treatment?

- Comprehensive characterization is needed of the roles played by acute and chronic senescence in renal homeostasis and disease—identifying when senolytic/senomorphic use will be safe and maximally effective.

### How Can We Detect If a Patient Is Responding to Therapy?

- Development of noninvasive tools to validate changes in senescent cell burden and efficacy of SC targeted therapies *in vivo* in patients (such as serum and urine biomarkers of senescence).

By understanding the role of senescence in kidney homeostasis and disease, developing new, targeted compounds and the tools to allow their efficacy to be charted noninvasively, it should become possible for senolytic treatments to move from the bench to bedside.
